# Lived Experiences of Newly Diagnosed Type 1 Diabetes Mellitus Children and Adolescents in Uganda

**DOI:** 10.2147/JMDH.S389265

**Published:** 2022-11-18

**Authors:** Jonathan Nsamba, Gloria Nabirye, Sibasis Hense, Fotios Drenos, Elezebeth Mathews

**Affiliations:** 1 Department of Public Health & Community Medicine, Central University of Kerala, Kasaragod, Kerala, India; 2 Department of Life Sciences, College of Health, Medicine and Life Sciences, Brunel University London, London, UB8 3PH UK; 3 Department of Nursing Sciences, Faculty of Health Sciences, Busitema University, Mbale, Uganda

**Keywords:** children, diabetes, perceptions, lived experiences, Uganda

## Abstract

**Introduction:**

The first-year post-diagnosis is the most challenging and stressful period in the lifetime of a young child and adolescent living with diabetes, given the adjustments that are meant to be adopted. Therefore, psychosocial factors affecting newly diagnosed children and adolescents need to be well understood and children supported to improve treatment adherence. However, evidence concerning psychosocial experiences among young patients with diabetes is scant in Uganda. This study explores the perceptions and experiences of newly diagnosed children and adolescents in Uganda.

**Methods:**

A qualitative exploratory design was employed. We recruited participants aged 6 to <18 years diagnosed within twelve months from three study sites: Mulago National Referral Hospital, Wakiso HCIV, and St Francis Nsambya Hospital. Twenty in-depth interviews were conducted, and textual data were analysed thematically using a framework approach.

**Results:**

We identified five themes: battling with symptoms, emotions at diagnosis, challenges in coping with diabetes management, changes I have made, and positive outcomes registered. The analysis found that young people living with diabetes experience a new world of adjustments, including insulin therapy, routine blood glucose monitoring, and dietary changes that are often difficult to deal with, especially in the first year after diagnosis.

**Discussion:**

Continuous psychosocial support to newly diagnosed young children and adolescents with T1DM is vital. Addressing psychosocial challenges may improve adherence to treatment regimens.

**Conclusion:**

Our findings have demonstrated the mixed experiences of newly diagnosed young children and adolescents living with diabetes, from anxiety and stigmatization to independence.

## Introduction

Globally, over 1.2 million children under 20 years are living with Type 1 Diabetes Mellitus (T1DM).^1^ T1DM, sometimes referred to as insulin-dependent diabetes mellitus, is caused by an autoimmune response in which the body’s immune system attacks the beta cells of the islets of Langerhans, resulting in an inability to produce sufficient insulin for metabolic functions.^2^ The International Diabetes Federation indicates that Africa is home to over 50,000 children living with T1DM, while in Uganda, over 3000 children and adolescents live with T1DM.^1^

Immediately after diagnosis, newly diagnosed T1DM children and adolescents face several changes in their daily life, such as frequent glucose monitoring, strict treatment, adherence to a meal plan, and exogenous insulin injections at the right time and dosages. This shift often results in psychosocial challenges of anxiety, depression, and stress.^3^,^4^ Published studies have indicated that T1DM patients have 2–3 times more risk of depression than healthy individuals,^5–7^ partly attributed to maladjustment in daily routines.

A recent systematic review^3^ has explored the prevalence of depression and anxiety among children with T1DM, standing at 22.2%. Stress and anxiety have been reported to decrease adherence to treatment among T1DM children and adolescents.^8–11^

There is some evidence indicating that the first year after diagnosis is the most challenging and stressful period during the life of a newly diagnosed child or adolescent^12^ due to changes in daily routines. The treatment of T1DM involves careful regulation of insulin therapy, daily monitoring of blood glucose, engagement in physical exercises, and diet modifications. To improve adherence and quality of life of young children and adolescents living with T1DM, psychosocial factors must be understood. Several studies have explored lived experiences, and quality of life among T1DM children and adolescents, mainly in high-income countries,^13–15^ but only a handful of studies are reported in low and middle-income countries; India,^16^ Tajikistan,^17^ Zambia^9^ Zimbabwe^18^ and Kenya.^11^ These studies enrolled young people, their parents and health workers and investigated their lived experiences and stressors about diabetes; however, none has exclusively studied the events that newly diagnosed children go through. Generally, studies exploring the lived experiences of newly diagnosed children and adolescents are limited, especially in developing countries. Therefore, this study explored the lived experiences, perceptions, and strategies to cope with the news of diagnosis through the lens of newly diagnosed Ugandan children and adolescents.

## Methods

The study explored the feelings and perceptions of newly diagnosed T1DM children and adolescents about diabetes, and examined the facilitators, and barriers to self-care by employing qualitative methods and an exploratory design.^19^ Specifically, this approach involved an investigation of the lived experiences of living with diabetes in its real-life context as described by the children and adolescents in Uganda. It allowed the authors to capture details of individual experiences in order to have an understanding of their views and perceptions regarding their disease.

From an interpretivism paradigm,^20^ this study conducted in-depth interviews to understand the individual experiences and contextualise the life experiences of T1DM patients. The study has followed the Consolidated criteria for Reporting Qualitative research, which allows a comprehensive reporting of findings from in-depth interviews.^21^

### Study Design

The study employed a qualitative method and a phenomenological design to understand the lived experiences of individual participants. The design allowed children and adolescents to share experiences in their own words as the research team examined the diabetes phenomena. Responses from the participants gave essence to their experiences, and the life stories shared informed an interpretive understanding of the diabetes phenomena. This study hinged on the model that one’s health status results from interactions between the individual and their social, economic, cultural, physical, and psychological markers. We used the model to explain that the child’s health status results from adherence to prescribed insulins and other medications, which in turn depends on their psychological and social well-being.

### Study Settings

The study was carried out in Uganda, an East African country. Participants were selected from diabetic clinics of Mulago National Referral Hospital, St. Francis Hospital, Nsambya, and Wakiso Health Centre IV. These hospitals were purposively sampled because they are some of the biggest in Uganda,^22^ with a high patient volume. Mulago National Referral hospital is located in Kampala Capital City, the country’s main Government owned hospital, with a patient caseload of over 200 T1DM children and adolescents. St. Francis Hospital Nsambya is a private, not-for-profit facility under the Uganda Catholic Medical Bureau, located in Kampala district, central Uganda. The hospital serves a large population of about 200 children and adolescents with T1DM. Wakiso Health Centre IV is Government owned and located in Wakiso district, central Uganda and has a total patient caseload of 40 children and adolescents. All the sampled hospitals are located in the central region of Uganda, an area predominantly characterised by western influence on diets and urban sprawl. The hospitals serve a diverse population beyond their neighbourhoods, villages or townships, and it is common for the patients to travel long distances to these facilities in search of specialised medical care.

### Sampling and Sample Selection

A sample of newly diagnosed children and adolescents under the Changing Diabetes in Children (CDiC^®^) program was recruited. Newly diagnosed referred to all patients who had been on insulin therapy for less than one year. The CDiC^®^ program is a public-private partnership between the Ugandan Government, Novo Nordisk, Roche, the International Society for Paediatric and Adolescent Diabetes, and the World Diabetes Foundation.^23^ The program aims to increase access to diabetes care for children living with T1DM by providing insulin, syringes, glucose monitors, and diaries for self-monitoring and recording blood glucose, among others.

Participants were children and adolescents aged 6- <18 years with clinically diagnosed T1DM, purposively sampled.^24^ Criterion purposive sampling^25^ ensured that only newly diagnosed (within twelve months) were enrolled. A sampling frame of only newly diagnosed children was obtained from the management of the CDiC program, and these were invited to participate in the study. This ensured that we obtained insights into the plight that newly diagnosed children face. We excluded patients out of the age range, those that did not consent, and those who had been in care for more than one year.

### Data Collection

The study utilised a semi-structured interview guide (Table 1) with main and probing questions to facilitate discussions.^26^ The first author [JN] conducted all in-depth interviews lasting between 45 and 60 minutes, assisted by one of the co-authors [GN]. Data collection was completed from March 2021 to January 2022. The sample size was determined after reaching the point of data saturation,^27^,^28^ where no new information regarding living with diabetes, challenges, and barriers to care were obtained. Twenty face-to-face, in-depth interviews were conducted in a location agreed upon by clinic management and the study team. A private room was made available for the interviews in all three study sites. Parents or caregivers were allowed to attend the sessions to offer emotional and physical support to the participants.

**Table 1:** In-Depth Interview Guide

Category	Questions
Discussion point 1	How did you react when you were told you had diabetes? ● Thoughts and feelings regarding the diagnosis ● How was the diagnosis process?
Discussion point 2	How do you feel on a day-to-day basis? ● Emotional and psychological wellbeing ● Social relations with family and friends ● Self-esteem among peers and schoolmates
Discussion point 3	What changes have you made in your daily routines due to diabetes? ● What psychosocial activities they engaged in ● Nutrition and physical activity changes
Discussion point 4	What challenges do you face in seeking treatment and managing diabetes? ● Psychological issues ● Costs related to transport and medicines
Discussion point 5	Are there any positive results you got experienced as you care for yourself? ● Experiences that are meaningful

Interviewers used open-ended questions to encourage participants to express themselves freely during interviews.^29^ The interviewer established rapport, which helped children feel at ease by making discussions fun, brighter, playful, and childlike.^29^ Field notes were taken during the interviews, and four repeat interviews (four respondents had a second round of interviews) were done to seek clarifications and additional information from participants of interest.

Since data collection was conducted within a time when the COVID-19 caseload was high, safety precautions were in place to guarantee the health and safety of research teams, participants and their families. There was routine temperature screening, the mandatory wearing of face masks, hand washing with soap and water and physical distancing throughout the implementation of research activities.

To ensure rigour and trustworthiness, the interview team had specialised training and experience conducting qualitative studies with children. They spent considerable time collecting data, including observing participants getting their insulin shots. After each interview, debriefing and double-checking of the content were carried out. Follow-up questions and probes were used to validate responses during the interviews. Dependability was ensured by having a clear audit trail and having two independent researchers running the analysis.

### Data Analysis

Audio recordings were transcribed and translated into the English language for those respondents that could not comfortably express themselves in English. Two people verified verbatim transcripts, including the first author (JN) and a co-author (GN), to ascertain that the transcripts were an accurate record of the audio recordings. Data were analysed thematically following the six steps of thematic analysis; transcription, reading and familiarisation, coding, searching for themes, reviewing themes, defining and naming themes, and writing up the results.^30^

Transcribed content and field notes written from the interviews were imported into ATLAS.ti 9 Windows, Scientific Software Development GmbH^31^ for coding. This iterative process involved codes being condensed into similar patterns and themes, using meanings from the participants’ responses. Coding was done to highlight insights into the participants’ experiences by analysing their textual words, and similar codes were grouped into themes. An analytic tree framework was developed with categories of similar codes condensed into patterns and themes. Lastly, we undertook an examination of patterns and themes to ensure that the analysis retained the original meanings and responses obtained from the participants.

## Results

The study explored the experiences of newly diagnosed type 1 diabetes mellitus children and adolescents in Uganda. A summary of the sample characteristics can be seen in Table 2.

**Table 2:** Participants’ Sociodemographic Profiles

Attribute	Frequency
Sex	
Male	08
Female	12
Age	
6–10 years	03
11–15 years	08
16–18 years	09
Study site	
St. Francis Hospital Nsambya	07
Mulago National Referral Hospital	10
Wakiso Health Centre IV	03
Place of stay	
Rural	06
Urban	14
Guardian/ Parents’ occupation	
Skilled work	05
Semi-skilled	08
Unskilled/ casual labourer	07

### Summary of Qualitative Findings

Here we present findings summarised as themes that emerged from the interviews. We identified five themes; battling with symptoms, emotions at diagnosis, challenges in coping with diabetes management changes I have made, and positive outcomes registered as summarised in Supplementary Table S1.

### Theme One: Battling with Symptoms

Participants had experienced several signs and symptoms before being diagnosed with T1DM; unfortunately, many of the respondents had been treated for other illnesses prior to diagnosis. Among the common symptoms that participants reported having experienced before diagnosis included constant headaches, fatigue, frequent urination and tiredness before diagnosis. Three participants reported constant headaches, two reported constant pains and body fatigue, one reported frequent urination, and three reported Malaria and Pneumonia before diagnosis. One of the respondents said,

I was always having frequent headaches, and I could not even concentrate on anything.

Male, 17 years old

I was told I had Diabetes last year in secondary class one. I remember I would always feel so weak and tired, and one day I fell sick while at school, and my mother took me to the hospital for a medical check-up. That is when the Doctor told me I had diabetes.

Male, 15 years old

Before being diagnosed with diabetes, some respondents had been falsely managed for other medical conditions. This is quite common in this part of the world, where there is a tendency for patients to seek medical care late after falling sick. One respondent said,

It took so long for my parents and medics to diagnose diabetes; I was always getting treatment for pneumonia and other associated diseases.

Female, 17 years old.

Similarly, another respondent said,

Just in a week, I got very ill and was admitted to a clinic where I was treated for malaria, but after two days without any change, we were transferred to a hospital where they removed my blood and later told me I was suffering from diabetes.

Male, 15 years old

In a health system where diabetes is mainly managed in big regional and national tertiary health facilities, late diagnoses are common. Unfortunately, in many such circumstances, patients present with deliberating conditions and complications.

### Theme Two: Emotions at Diagnosis

**Figure 1:**
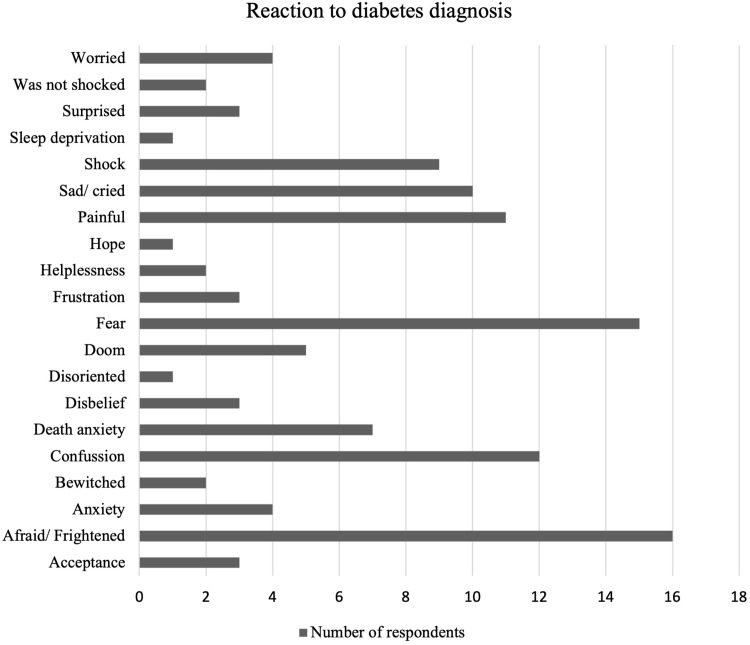
Nature of emotions experienced by participants to a diabetes diagnosis.

Most respondents reported mixed reactions to the news confirming diabetes diagnosis, from fear, confusion, and anxiety to being unsurprised and unruffled. Many participants were visibly sad when asked to share what and how they felt the moment they got informed of the diagnosis. Figure 1 summarises the different emotions that respondents voiced.

#### Subtheme I: Scared and Confused

When asked about their reaction when they were told they had diabetes, sixteen children reported being afraid and frightened. One of the respondents said,

At first, I did not understand what he meant by diabetes until he translated it as ‘Sukaali’. This made me feel anxious and terrified because I had a relative who had succumbed to ‘Sukaali’. I was also scared of the treatment because the doctor said I was supposed to inject myself, yet I used to fear injections.

Male, ten years old.

Another respondent also reported,

Despite this, the confirmation of my diagnosis wasn’t simple to deal with because I feared the daily injections I have heard about!

Female, 11 years old

**Figure 2:**
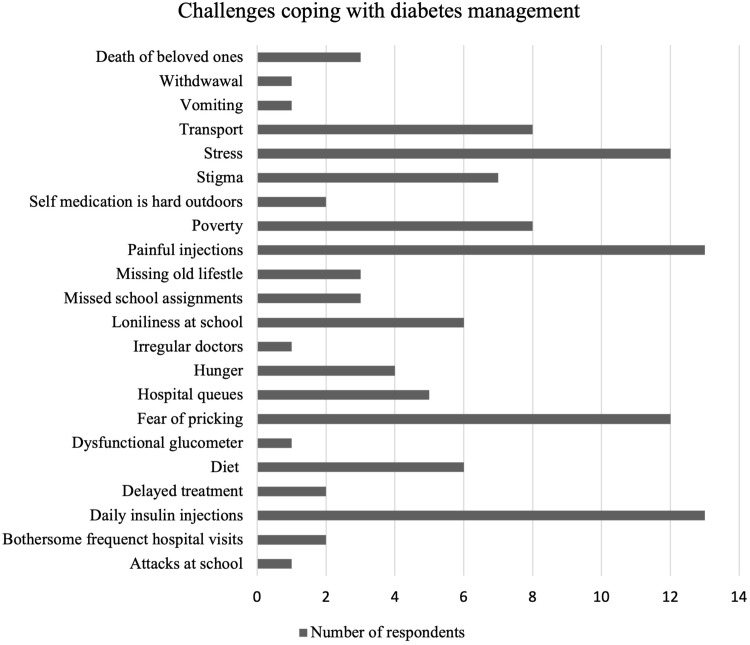
Challenges in coping with diabetes management.

Some of these respondents received the news with fear and confusion partly because of what they had previously been told about diabetes. Culturally, there is a common fear of chronic diseases with many cultural superstitions, such as being bewitched. Secondly, participants had prior knowledge concerning daily injections, which young children usually dread. It looked clear that most of the participants’ main fear was the daily shots of insulin due to the anticipated pain from previous injections experiences (Figure 2).

I was scared because what I was told meant I would have to be taking medicine almost every day, and there is no cure.

Female, 14 years old.

Some children said it was hard to accept that they had diabetes

I was scared of how I’ll deal with it. So, it wasn’t easy to take in.

Male, 17 years old.

Twelve respondents reported being confused after being told they had been diagnosed with Diabetes. One of the respondents said,

I was confused; I did not know what would happen to me after this. I did not know how I was going to tell my friends about my condition, and I was also scared of how they would react.

Male, 10 years old.

Another respondent said she did not know what to do after being told the diagnosis;

Once I was diagnosed, I did not know what to do.

Female 17 years old.

Emotions at diagnosis of fear and confusion were reported to manifest as tears. Ten respondents were able to illustrate that they were very sad, cried and felt worried. Respondents mentioned how low the news of the diagnosis made them feel. One said,

I cried. I felt bad when I was told I had diabetes.

Female, 14 years old.

I felt so bad and thought my life was coming to an end.

Female 17 years old.

I wondered why me. I wondered why I had to get such a big unending condition.

Female 17 years old.

Some respondents said they were shocked that diabetes also affects young children. They had a premise that diabetes is for adults and not children. One of the respondents said,

My family and I were shocked by the news, and I could not myself truly believe I had diabetes. No one in my family has it.

Male, 12 years old.

This is a disease I always heard terrible things about. I felt like there’s no way that I am diagnosed with it, that maybe it should’ve been an error of some kind.

Female, 17 years old

I thought young children do not suffer from such diseases.

Male, 16 years old.

I was diagnosed with Diabetes at a young age. This was at a medical camp organised by the church community in my area of residence in Zana. I had never known any of my age mates with this condition. This scared me. However, the doctor later explained to me that diabetes is also common among children and that I wasn’t the only child with the condition

Female, 07 years old.

Such reactions of fear and confusion indicate the negative feelings that young children and adolescents experienced at the time of diagnosis. We observed that there was a psychologist present in the clinic to offer counselling and psychosocial support; however, not all children were seen by the professional. Only those battling with poor adherence, evidenced by their glycated haemoglobin levels and missed appointments, were referred to meet the counsellor.

#### Sub-Theme II: Fear of Death

Some respondents reported fear of death after diagnosis. One of the respondents said,

I was scared of death; I wondered why me.

Male, 15 years old.

I was told when you get diabetes, you can die without knowing when you are sleeping. I was always scared of sleeping at night.

Female 14 years old

To other children, the fear of death was attributed to having heard of or witnessing their beloved ones dying because of diabetes. One of the respondents said.

Because my father died, I also started thinking I was going to die.

Female, 17 years old.

Similarly, a respondent said,

I felt like I was going to die just like her.

Male, 10 years old.

We observed that young children and adolescents had similar thoughts regarding death from diabetes. It would be expected that adolescents are much more aware, but it was not the case as they were too afraid of death. During the time of data collection, Uganda was experiencing a second wave of Covid-19 disease. The population had been informed that people with diabetes have an increased mortality rate from Covid-19 than those without diabetes. One of the adolescents reported being worried and scared of COVID-19

I am afraid of COVID-19 because, on the news, they said that people with diabetes easily die first.

Female, 17 years

### Theme Three: Challenges in Coping with Diabetes Management

Participants’ critical challenges in everyday life were classified into broad categories of self-care, psychological aspects, school environment, diet therapy, and access to medical care and supplies. Figure 2 summarises the number of respondents who experienced a particular challenge.

#### Sub-Theme I: Stigma and Stress

Seven children reported having experienced stigma from their friends and classmates, making them uncomfortable, especially at school. The most stigma experienced was from the premise that T1DM was contagious. Other students did not want to associate with those known to have diabetes as a way to avoid getting infected.

One of the respondents said,

Maybe the school part, it’s one reason I actually hate school, everyone including all teachers, just have to be told you are diabetic.

Male 17 years old. Considering this respondent’s reaction, stigma was cultivated both by fellow peers and also school staff.

Similarly, another respondent said,

Some children at school don’t play with me because they think that I will spread the disease to them.

Male 17 years old.

I remember that time was not easy. I went back to school, and my friends were scared, thinking I was going to infect them. But thank God this did not happen.

Female. 12 years old

It is important to recognise that these children spend most of their time in school, which is the same environment in which they are discriminated against and stigmatized. We found out that even outside their schools, they still faced stigma. One of the respondents reported experiencing stigma from hospitals as they come for their appointments and medical checkups. It was said,

Not that it’s like HIV, because we are told about HIV at a young age, unlike diabetes, but at some point, they will shout and address you as ‘hey, you with diabetes…’ in front of everyone

Male, 17 years old.

It was evident that how society responds towards these young children profoundly affects their self-esteem and well-being. It was interesting to learn of the gaps that exist in school settings, especially regarding stigma. It is important to appreciate the critical roles that school staff can play in diabetes management, from supporting children with their insulin shots to ensuring exercise and nutrition therapy. One of the respondents said,

When I am free, I take a walk at the school campus to burn some calories as advised by the school nurse, whom I visit regularly.

Female, 16 years old. Another participant said,

At school, my mother paid for some special food for me. I get to have at least a banana or an apple every day as a source of vitamins.

Female, 16 years old.

Key school staff like teachers and school nurses have undeniably essential roles to play as far as the children’s physical, social and psychological well-being is concerned.

#### Sub-Theme II: Will the Injections Ever End?

Some of the respondents had apparent fatigue related to the daily injections. This was attributed to the pain that is associated with the injections. We witnessed a health education session where the nurse informed the children that there was a low grade of pain related to the shots due to their small nibbles, but still, the children feared them. Some respondents reported that they felt terrible about the daily medications and injections.

Sometimes I feel like the insulin injections are a big problem.

Female, 07 years old

Managing diabetes takes a lot of effort and not forgetting. Daily injections are unpleasant and tough to overcome. Sometimes, I get abrupt mood swings.

Male, 12 years old

The part where I inject myself has become very hard, and the scars are painful and look bad.

Male, 16 years old

I do not feel very well because of the daily injections. I always feel bad every day as if God left me.

Male, 16 years old

Another respondent reported disliking the fact that diabetes has no cure

I dislike the injections and would much prefer a cure, but ever since I understood that this is something that’s not going away, I am trying my best to feel the way my parents want me to feel

Female, 17 years old.

Another child respondent reported being uncomfortable with the burden of daily medications

The feeling of being reminded every day to take medicine makes me feel uncomfortable.

Female, 14 years old.

#### Sub-Theme III: Stressors

Respondents voiced their concerns over moments when they felt stressed. Some children reported that it was stressful, frustrating and hard to live with T1DM. One of the respondents said,

Honestly, I have been completely miserable ever since the diagnosis.

Female, 17 years old

Another added,

Every time I am worried about tomorrow. I feel stressed and sticking to diabetes is very difficult for me.

Female, 13 years old.

One of the female respondents said,

Mentally, I feel tired about living each day. Sometimes I forget to take my medicine.

Female, 14 years old.

I wish I did not have it such that I live like other friends of mine who do not mind about anything.

Female, 17 years old

To some, the frustration was primarily due to the life adjustments they were meant to live by. These restrictions were sometimes imposed by their parents and caregivers to prevent any resulting adverse event, such as an acute blood glucose imbalance. Unfortunately, children do not see it with the same lens. One of the respondents reported,

I feel bad because I am limited to what I am able to do. I cannot play as much as I would want.

Female, 13 years old.

To another respondent, the idea of being restricted in food choices caused stress. She said,

I feel bad because I do not eat everything that the rest are eating, especially if it may have a lot of sugar.

Female, 17 years old.

Sometimes I get frustrated because of the restrictions on which foods I should avoid, for example, when I want ice cream but cannot take it. I am always scared that my condition could worsen at any time.

Male, 10 years old

Several respondents reported experiencing long waiting times due to long queues when they came for their hospital appointments. We observed that most children arrived at the clinics between 8 and 9 and left at midday. One of the respondents said,

Sometimes you come to the hospital and end up waiting for a long time.

Male, 10 years old

One further stated that sometimes the health workers are late;

Sometimes the doctor responsible for attending to us comes late.

Male 12 years old. Similarly, a young male said,

Sometimes I miss school because I have to go for checkups.

Male, 10 years old.

Respondents highlighted the high transport fares being one of the major hindrances. One of the respondents said,

Transport is also a big problem really coming to the hospital from Kajjansi to Nsambya. That is why sometimes I miss coming to the clinic.

Male, 16 years old.

Similarly, another respondent said,

Sometimes I don’t have transport to come to the hospital every two weeks.

Male, 17 years old. Another indicated,

Sometimes my parents do not have the money for transport.

Female, 07 years old.

Another respondent reported that their family had thought it was witchcraft.

When the doctor told me that the disease cannot be cured, I was surprised. My mother thought I had been bewitched, so she took me to my auntie, who prayed for me. My aunt said I was cured, but I still had frequent headaches. So, my mother brought me to hospital again

Male, 17 years old.

These situations are challenging for any young child to bear and live through. Stressful thoughts and moments have a short and lifelong effect on emotional, psychological and social well-being. These sources of stress act in the immediate and surrounding environment in which these children and adolescents live, from homes, schools and hospitals.

#### Sub-Theme IV: Lack of Food and Medical Supplies

Children living with diabetes are encouraged to have access to a well-balanced, healthy diet that follows the recommendations provided to them by their dietician. However, in a society with a high cost of living, access to safe and nutritious food is sometimes challenging. Six respondents indicated that the high food costs and poor access to healthy and nutritious foods continue to be difficult.

I do not access fruits and vegetables which we are told to eat.

Female, 17 years old.

Another respondent said,

I inject myself and then eat in 30 minutes after the injection if I have food. Unfortunately, sometimes when I do not have food, I have to inject myself but do not eat afterwards

Female, 17 years old.

My diet is mainly made up of posho and beans. I usually do not eat enough due to lack of food.

Female, 14 years old.

For these respondents, access to healthy foods is a challenge, which is a risk factor for hypoglycaemia if the participant injects insulin. Regarding the lack of medical supplies, only one respondent indicated a lack of a functional glucometer for blood glucose measurement.

My glucometer stopped working; I now only measure when I come here at the hospital because my glucometer stopped working.

Male 10 years old.

Access to medical supplies such as syringes, insulin, glucose monitors and testing strips is not a common challenge to this group of children, given that the CDiC program provides them.

Three respondents expressed their dissatisfaction with their parents over the use of herbal medicines. One of them indicated, “When I am dizzy, my grandmother gets ‘mululuuza’ (local herb) and ‘Kigaaji (local herb)’ for me” I am not sure they work. Female, 07 years old.

### Theme Four: Changes I Have Made

#### Sub-Theme I: Diet and Eating Habits

The respondents reported having made changes, in their daily living, especially with matters pertaining to food and nutrition. We observed the presence of a dietician who gives dietetic advice and support to young children and adolescents. Responses from participants indicated a good understanding of dietary approaches in diabetes. One of the respondents said,

But I now watch what I eat. I do not eat Rolex (a local snack) and Soda. We were told to avoid Soda because it increases our sugar levels.

Female, 17 years old.

The first thing is, I was fat, but now I am small. I was very fat, like a broiler chicken, but now I feel fitter. I stopped taking soda; … Oooh, Mirinda fruity. I loved it a lot, but now I stopped taking it

Female 16 years old.

Another responded,

I always make sure that the food I eat has less sugar.

Male, 12 years old.

These respondents showed a good level of understanding regarding the vital dietary principles of diabetes. However, we also understood that knowledge and practice are different. Due to factors beyond the participants, access to food might be tricky.

It was interesting to find out that respondents knew the time to eat in relation to their medication, yet they had just been on the program for less than one year. One of the respondents said,

I also have to eat one hour before checking my glucose levels.

Male, 17 years old.

Furthermore, another respondent emphasised the role of parents in ensuring an adequate diet even when the child is in a boarding school.

At school, my mother paid for some special food for me. I get to have at least a banana or an apple every day as a source of vitamins.

Female, 17 years old.

#### Sub-Theme II: Adherence to the Treatment Regimen

Adherence to the prescribed insulin treatment is essential for glycaemic control. Since most respondents were school-going, we needed to understand whether they adhered to therapy at school and home. From the responses, some participants pointed out that they had adapted well to taking their daily medications and having regular medical check-ups.

I take my daily injections of insulin which I schedule every afternoon, which is nearly the same time as I have lunch. Every two weeks, I visit the hospital to let the doctors and nurses check on me

Female, 17 years old.

It was interesting to note that some young respondents have grown to take control over their health, from depending on their parents to them now making decisions. One of the respondents said,

I have also learnt how to do the calculations for my insulin treatments. Previously my dad used to help me with the pricks, but now I can prick myself.

Female 11 years old.

The majority of the children stated that they had increased their deliberate attempts to engage in everyday exercises as taught during clinic days.

I now do some exercises, especially crunches and rope skipping. They help me sweat and are a good physical activity. After sweating, I shower and then I feel good. I was told to do physical exercises every day for the insulin to work well.

Female, 14 years old

Because of diabetes, I started regular exercises by participating in co-curricular activities at school; it is always emphasised.

Female, 17 years old.

One of the adolescents said,

When I am free, I take a walk at the school campus to burn some calories as advised by the school nurse.

Female, 17 years old.

I have also learnt how to do some exercises because of diabetes.

Female 11 years old. Another one said,

I exercise as part of my diabetes care.

Male 12 years old.

### Theme Five: Positive Outcomes Registered

**Figure 3:**
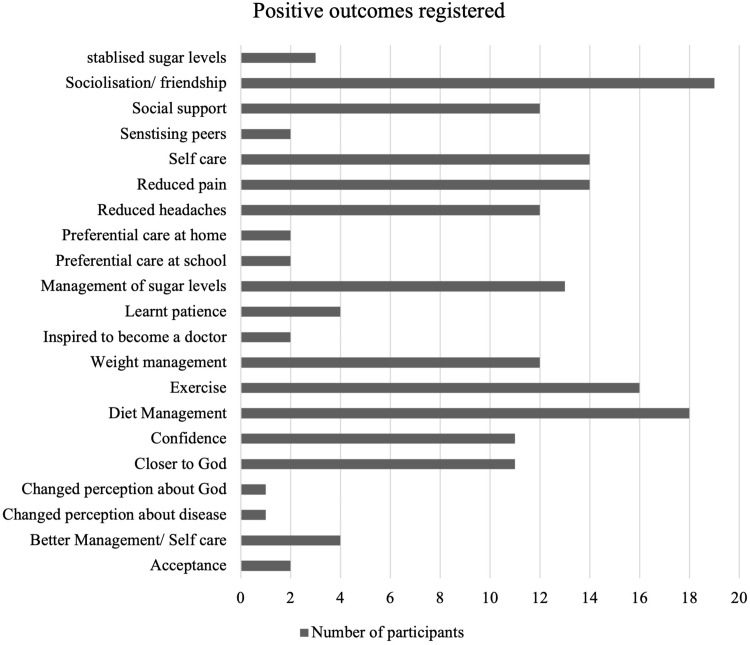
Positive outcomes registered.

Amidst the challenges that respondents reported, some reported having adjusted well to living with diabetes. Feedback from respondents concerning positive outcomes resulting from diabetes treatment and management involved the feeling of maturity, independence and enjoying preferential treatment. It was observed that most of these respondents that had adjusted better were older adolescents. This was attributed to the education that they had obtained over time. Additionally, social groups offered a sense of belonging and a forum where they could share and relate. Figure 3 shows the number of participants who experienced a given positive feeling or experience.

One respondent said,

After all these months of living with diabetes, I can now say that I am well. I have learnt to live positively, and I have accepted that it is a life condition I have to manage. I am more aware of when I am going to get into a crisis.

Male, 15 years old

Another respondent also said,

I now feel more confident, unlike before when I was always afraid of pain from injections. I was usually sick last year, but now it is like my body is now getting used to the disease.

Female, 17 years old.

I also know that this condition is manageable; that way, I can focus on other aspects of my life that matter, like my studies.

Female, 17 years old

Another respondent indicated that they had gained hope through their other family members who also have diabetes. This showed the vital role that families play in acceptance, adherence and fostering a positive outlook.

On a day-to-day basis, well, I have grown up the only child who is diabetic among my sisters and brothers; no one takes the same medicines I take at home, not even my parents, except I have also grown up knowing my grandmother and my aunt who is also diabetic. So, I have seen them survive, and they are happy; I have not really stressed over it because they told me it’s only about managing and controlling my blood glucose levels

Male, 17 years old. Some participants, as illustrated in Figure 3, expressed their pleasure in being able to care for themselves. This was attributed to children’s capacity-building and health education sessions during clinic visits. We observed a health education session facilitated by the study nurse and dietician.

One of the respondents said,

I manage my body better now, I remember how I suffered last year, but over time, I have learned how to eat and live. I am better. I hope I can heal for good.

Female, 17 years old

Another one said,

I now manage my diabetes better due to the health education we have got from here in the hospital. I have also learnt how to take care of myself.

Male, 09 years old

It is worth noting that these young people living with T1DM are trying to adhere to the science and art of their treatment and are on the right course to independence. One said,

I have learnt to manage my blood sugar levels. I get to know when it is getting out of normal range and how to intervene, aiming at not having any of those bigger complications that they had told me about

Male, 17 years old.

The presence and friendships from peers and social support provided by social workers and counsellors have increased these children’s confidence. One female adolescent said,

I am no longer scared of anything. I have also met other children in the hospital who are like me, and we have become friends.

Female, 17 years old.

Another said,

I used to be very quiet, but now I feel happier because I get to see other people apart from my relatives. It has made me get to know fellow sick people.

Female, 16 years old.

I have friendships with fellow patients and nurses here, which is helpful and a source of information about diabetes. This has also enabled me to assist my other friends to help them understand that diabetes is not a death sentence

Female, 13 years old.

## Discussion

We aimed to explore the lived experiences of children and adolescents newly diagnosed with T1DM in Uganda. Key findings from the interviews indicated that children faced negative and positive experiences from stress, anxiety and depression after diagnosis to adherence, independence and progressive improvement over time. Several factors, such as stigmatisation, and daily insulin injections, were partly responsible for the adverse reports of stress and anxiety. The benefits and positives reported by the participants, such as diabetes literacy, care for oneself and support groups, are attributed to the health education, counselling and capacity-building sessions the participants get during their routine clinic visits.

Findings revealed that most children, caretakers, or parents were unaware of the cardinal signs and symptoms of T1DM, especially before diagnosis. Similarly, some health workers would not rightfully diagnose diabetes which largely contributed to the late diagnoses reported by the participants. The lack of capacity to rightfully identify, diagnose and manage diabetes in developing countries is still a public health challenge. Integrating diabetes management and care across all levels of Uganda’s health system will be vital in fostering active and timely case finding.

Participants stated that at diagnosis, they had suffered episodes of psychosocial effects, especially shock and fear. It was observed that both children and adolescents had experienced similar experiences of fear and shock at diagnosis thus, the age at diagnosis did not seem to influence the nature of feelings. Similar results regarding initial feelings of shock, fear and tears were observed in Turkey,^32^ Zambia^9^ and Kenya.^11^ While we observed the presence of a counsellor during the clinic day, not all newly diagnosed patients were referred for psychosocial support. Only those that were noncompliant with the treatment regime would be referred. The criteria for referral were low adherence, many missed appointments and high glycated haemoglobin levels. This underscores the need to counsel all new patients and manage stress right from diagnosis.

Similar findings of depressive symptoms and anxiety at diagnosis were reported by other studies.^6^,^33^,^34^ Participants found diabetes management stressful and challenging to cope with, partly due to the enormous demands as far as T1DM management is concerned.^35^ It was observed that several participants had experienced stigmatisation, especially at school or social gatherings, mainly by friends, peers and classmates. One of the main reasons for stigmatisation was the false premise that T1DM was contagious. This was similarly observed among Zambian children,^9^ who also faced stigmatisation from peers. There is a need to raise awareness of T1DM among schools and the wider public to break this myth, especially in Uganda, given the cultural superstitions carried with chronic diseases. When young people with diabetes face stigmatisation from peers, they lose their sense of belonging, negatively affecting their psychological well-being.

We observed good child-parent relationships where parents were actively involved in the care of their children. Some studies have documented positive outcomes regarding parental involvement in diabetes care; for instance, parents reminded young T1DM children to take their medicines.^14^,^35^ While this is a positive outcome, there were instances where we observed restrictions against play and social engagements^35^ since they are regarded as “sick”. Such restrictions exacerbate stress and depression levels, and thus the role of parents and family needs to be carefully evaluated.^36^ Therefore, it is vital that more support and attention be given to the younger children and then gradually adjusted as they age so that a sense of independence is cultivated.

We observed that some participants had not adjusted fully to the recommended changes of positive living, including insulin therapy, diet, and physical activity. There was a need for medical practitioners to provide individually tailored support and counselling to such children and pay particular attention to the emotional and psychosocial challenges affecting them. Only then can they adhere to the treatment regimen that is prescribed.^37^

The study findings revealed that some participants were not happy with the quality of service being offered during medical visits. Some participants were unsatisfied with the long waiting time since they come early without eating. As similarly reported,^38^,^39^ people with diabetes are a particular case of patients who come without eating in preparation for fasting glucose measurements. This, therefore, requires that they spend the least time possible during routine medical procedures.^40^

It was realised that our participants had experienced episodes of death anxiety after learning that they had been diagnosed with T1DM. This was partly attributed to previous experiences of loved ones dying from the same disease. Additionally, many children suffered from death anxiety during the COVID-19 pandemic because of the high fatality rates reported among people with chronic conditions.^41^ Similar observations were made among youths and young adults with T1DM showing an increased risk of suicidal and death ideations.^42^,^43^ It is, therefore, essential that anxiety is addressed among young people with T1DM. This can be done by adopting an integrated care model that encompasses social, emotional, physical and psychological well-being, encouraging routine screening of depression, counselling and boosting health workers’ training to recognise and address psychosocial challenges.^42^

Among critical barriers to care was the lack of transport to come for routine medical appointments. While the direct medical costs associated with insulin therapy, testing strips, glycated haemoglobin tests and glucose monitoring devices are met by the CDiC^®^ program,^44^ indirect costs like transport remained a considerable burden to the families of these children given that most of the parents to our participants had semi-skilled or casual sources of income. In an already economically fragile citizenry, even transport costs are beyond many families’ means.^45^

It was also observed that herbal medicines were used by some respondents to treat diabetes. These respondents indicated that their parents and close family members provided these herbs as complementary therapy. The use of concomitant medications jeopardises the efficacy of the prescribed treatment regimen. Results reported in Zambia showed a similar occurrence of the use of herbal medicines.^9^ Therefore, this needs to be addressed, especially in Uganda, by educating and supporting families so that parents and caregivers understand the need to adhere to the prescribed T1DM treatment. The scientific community and medical practitioners are tasked with finding ways of supporting newly diagnosed young people with T1DM to cope with the new disease and changes in daily routines. Unless supported, we may fail to address psychosocial factors that can easily lead to undesirable coping strategies like avoiding insulin injections.^9^ Several approaches can be adopted to offer individually tailored support. For instance, emphasising the need for individual therapy sessions, counselling sessions for all newly diagnosed children, routine assessments and monitoring for diabetes-related stress.

Key strengths of this study include a deliberate attempt to document children’s voices that are often not heard. Many studies engage parents, caregivers and health workers without involving the young children that live with the disease. To the best of our knowledge, this is the first qualitative study to explore the lived experiences of newly diagnosed T1DM children and adolescents in Uganda. Among the limitations, the views expressed in this study lack generalisability toward the entire young population living with diabetes in Uganda. Lastly, the sampled participants were recruited from diabetic clinics and, as a result, might have different views and health-seeking behaviours from those who did participate.

## Conclusion

Findings from our study illustrate that among our participants, diabetes had a negative impact on psychosocial and emotional well-being. The news concerning the diagnosis was received with fear and disbelief by the children, parents and the entire family. These children need to be supported emotionally and psychologically to foster adaptation and adherence to the disease and its treatment.

Our children and adolescents reported facing a diversity of barriers to care, such as stigma, poor access to food and high transport costs. Medical practitioners in Uganda need to address each barrier to improve the quality of life and mitigate psychosocial outcomes such as stress, anxiety, and depression. Although we lack generalisability of our findings beyond the study population, the findings give an insight into the plight that young children and adolescents might experience. Our results constitute evidence that psychosocial problems are common among our sample of young children and adolescents living with diabetes in Uganda and, if not addressed, can progress and develop into diabetes-related distress. Based on the multidimensional WHO’s definition of health,^46^ it is vital to routinely examine these young patients’ physical, social, and psychological well-being as part of the medical management of T1DM.

## Data Sharing Statement

The data is not publicly available in line with confidentiality and privacy protection but is available from the corresponding author upon reasonable request.
